# Regulation and function of adiponectin in the intestinal epithelial cells in response to *Trichinella spiralis* infection

**DOI:** 10.1038/s41598-023-41377-x

**Published:** 2023-08-27

**Authors:** Siranart Jeerawattanawart, Adithap Hansakon, Sittiruk Roytrakul, Pornpimon Angkasekwinai

**Affiliations:** 1 Department of Medical Technology, Faculty of Allied Health Sciences, Thammasat University, Pathum Thani, 12120 Thailand; 2 Graduate Program in Biomedical Sciences, Faculty of Allied Health Sciences, Thammasat University, Pathum Thani, 12120 Thailand; 3 Chulabhorn International College of Medicine, Thammasat University, Pathum Thani, 12120 Thailand; 4 Functional Proteomics Technology Laboratory, Functional Ingredients and Food Innovation Research Group, National Center for Genetic Engineering and Biotechnology, National Science and Technology Development Agency, Pathum Thani, 12120 Thailand; 5 Research Unit in Molecular Pathogenesis and Immunology of Infectious Diseases, Thammasat University, Pathum Thani, 12120 Thailand

**Keywords:** Immunology, Microbiology, Infectious diseases

## Abstract

Besides metabolic homeostasis regulation, adipokines are recently emerged as important players in regulating immunity and inflammation. Helminth infection has known to modulate circulating adipokine secretion; however, the regulation and function of adipokines in response to helminth infection is still unclear. Here, we investigated the regulation and function of adiponectin during *T. spiralis* infection. While there was no change in circulating level of adiponectin, we found an increased adiponectin, but not leptin expression in the small intestine. Interestingly, the intestinal adiponectin expression was strongly associated with the expression of epithelial cell-derived cytokines IL-25, IL-33, and TSLP following infection. Indeed, mice deficiency of IL-25 receptor exhibited no intestinal adiponectin induction upon helminth infection. Interestingly, IL-25-induced adiponectin modulated intestinal epithelial cell responses by enhancing occludin and CCL17 expression. Using LPS-induced intestinal epithelial barrier dysfunctions in a Caco-2 cell monolayer model, adiponectin pretreatment enhanced a Transepithelial electrical resistance (TEER) and occludin expression. More importantly, adiponectin pretreatment of Caco2 cells prevented *T. spiralis* larval invasion in vitro and its administration during infection enhanced intestinal IL-13 secretion and worm expulsion in vivo. Altogether, our data suggest that intestinal adiponectin expression induced by helminth infection through the regulation of IL-25 promotes worm clearance and intestinal barrier function.

## Introduction

Helminths are parasitic worms that have co-evolved with the human host for a long time and affect people worldwide, especially in developing countries^1^. Intestinal epithelial cells function as the natural barrier against helminth invasion and the first responders that contact parasitic helminths. Upon helminth infection, various types of alarmin cytokines, including IL-25, IL-33, and thymic stromal lymphopoietin (TSLP) were secreted from intestinal epithelial cells^2–5^. These epithelial cell-derived cytokines strongly activated many cell types such as ILC2, Th2 cells, and mast cells to enhance the production of type 2 cytokines, including IL-4, IL-5, IL-9, and IL-13 that are critical to promote parasitic worm clearance^6–8^.

Accumulating evidence shows the association between helminth infection and a lower incidence of metabolic syndromes and suggests that host immune response induced by helminth parasite might regulate metabolic homeostasis^9,10^. Type-2 immune responses not only function to promote host immunity to helminth infection, but have been known to protect against metabolic disorders such as obesity, type 2 diabetes, cardiovascular disease, and atherosclerosis^11–14^. Previous studies indicated that infection with *Trichinella spiralis* improved insulin sensitivity, enhanced glucose tolerance, and reduced body weight gain by promoting M2 macrophage polarization^15,16^. Obese mice infected with *Schistosoma mansoni* had reduced body weight gain and enhanced insulin sensitivity through the induction of M2 macrophages and IL-33-induced group 2 innate lymphoid cells^17^. *S. mansoni* soluble egg antigens (SEA) were found to modulate metabolic homeostasis in obese mice through a STAT-6 dependent mechanism^18^. Similarly, obese mice infected with *Nippostrongylus brasiliensis* had enhanced eosinophils, type 2 cytokine-associated gene, and M2 macrophage gene expression in adipose tissue, liver and small intestine^19^.

Recent studies indicated that helminth infection may exert host immune response by not only modulating metabolic homeostasis but also directly regulating adipose tissue function^13,20,21^. Infection with *Strongyloides stercoralis* can deregulate the secretion of adipokines, mediators derived from adipose tissue, including leptin and adiponectin in children^22^. Furthermore, infection with *T. spiralis* reduced circulating leptin level that was associated with hypophagia and weight loss^23^. Compared with uninfected subjects, patients infected with helminth parasite *Taenia taeniaformis* had reduced leptin levels^24^. The increased ratio of leptin and adiponectin was associated with the removal of soil-transmitted helminth infections^25^. Recent study has demonstrated the increased anti-inflammatory adipokines, adiponectin and adipsin in plasma of patients infected with *S. stercoralis* was associated with the enhanced type 2 cytokine production^26^. These studies emphasize the possible involvement of adipokine regulation by helminth infection.

Besides the regulation of metabolic homeostasis within adipose tissue, adipokines have additional roles in modulating immune function. Adiponectin was known to suppress M1 macrophage activation^27,28^, promote M2 macrophage polarization^28,29^, maintain intestinal homeostasis^30^ and control pro-inflammatory CD4^+^ T cells^31^, whereas leptin was reported to promote Th1 immune response^32^, enhance natural killer (NK) cell cytotoxicity^33,34^, promote granulocyte^35,36^, macrophage^37,38^ and dendritic cell function^39,40^. Although the association between helminth infection and altered adipokine production has been suggested, the involvement of adipokines and its function during helminth infection remains elusive. In the present study, we investigated the expression of main adipokine adiponectin and leptin in response to *T. spiralis* infection and further elucidate the role and regulation of adiponectin in immune response to helminth infection. We found the upregulation of adiponectin expression in the small intestine of *T. spiralis*-infected mice at 7 days post-infection. During *T. spiralis* infection, IL-25 was shown to be the key regulator for the induction of adiponectin within intestinal epithelial cells. The direct role of adiponectin in promoting epithelial responses was observed in either in vitro models of LPS stimulation or larval invasion*. *In vivo treatment with adiponectin enhanced worn clearance and intestinal IL-13 secretion. Thus, our data revealed the crucial role of adiponectin in mediating intestinal response to helminth infection.

## Results

### *Trichinella spiralis* infection induced adiponectin expression in the intestinal tissue

**Figure 1:**
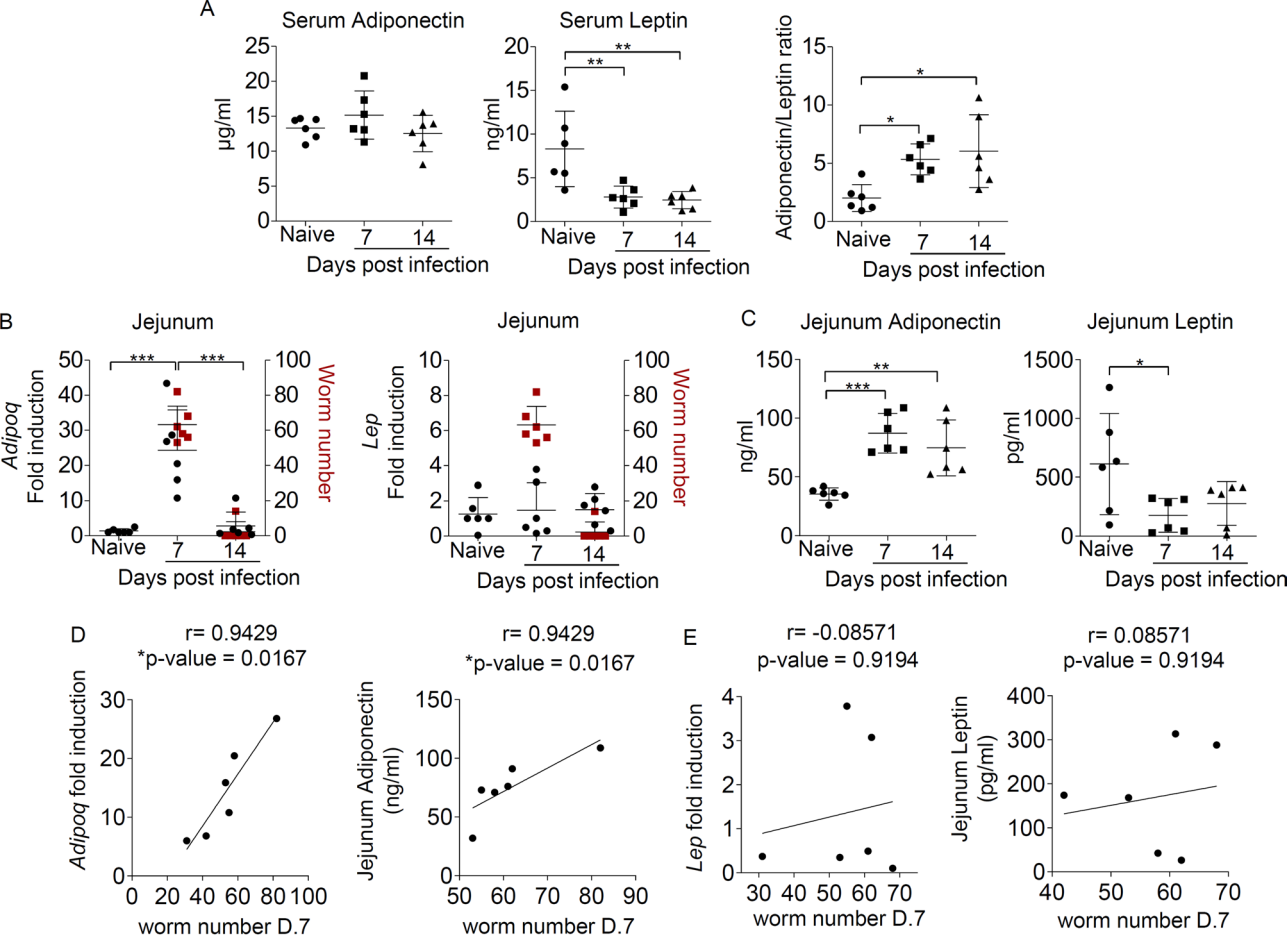
The induction of adiponectin in small intestinal tissue during *T. spiralis* infection. *T. spiralis* larvae were orally gavaged into BALB/c mice. Blood and small intestinal tissue were harvested at 7 and 14 days after infection to evaluate adiponectin and leptin expression using ELISA analysis and quantitative real-time PCR. (**A**) ELISA analysis of adiponectin, leptin, and the adiponectin/leptin ratio in serum from uninfected mice or mice infected with *T. spiralis*. (**B**) Quantitative real-time PCR analysis of adiponectin and leptin and the representative data of worm number in the intestinal tissue from uninfected mice or mice infected with *T. spiralis*. The mRNA expression data are presented as fold induction over actin (*Actb*) expression, with the mRNA levels in uninfected mice set as 1. (**C**) ELISA analysis of adiponectin and leptin in jejunum lysate from uninfected mice or mice infected with *T. spiralis*. (**D**,**E**) The correlation between intestinal worm number and the mRNA and protein expression levels of adiponectin (**D**) and leptin (**E**) in the intestinal tissue from mice infected with *T. spiralis* for 7 days. Graphs depict individual mice and mean ± SD is representative of three pooled independent experiments, with n = 6 mice per group. Significance was determined using one-way ANOVA followed by Turkey post hoc analysis and Spearman’s rank test. Correlation coefficients (r) and *p* values are provided. (*p < 0.05, **p < 0.01, ***p < 0.001).

Recent findings indicated that infection with intestinal helminth altered the level of adipokines^22–24,26^, suggesting the link between the regulation of adipokines and helminth infection. In an attempt to determine the involvement of adipokines during gastrointestinal helminth infection, we examined the expression of adiponectin and leptin in the serum and intestinal tissue of mice infected with *T. spiralis* at 7 and 14 days post-infection. Compared to the uninfected mice, we observed no significant difference in circulating level of adiponectin during infection, but a significant reduction of leptin level in serum of *T. spiralis*-infected mice at 7 and 14 days post-infection (Fig. 1A). Because the ratio of adiponectin and leptin have been proposed as a marker of adipose tissue dysfunction^41,42^, we enumerated the adiponectin/leptin ratio and detected a significant induction of the adiponectin/leptin ratio in serum of *T. spiralis*-infected mice at 7 and 14 days post-infection (Fig. 1A).

In addition to adipose tissues being the main source of adiponectin and leptin production^43,44^, the intestine has been reported as another tissue that expressed adiponectin and leptin^45–48^. Because *T. spiralis* larvae initiates infection by penetrating the epithelium of the small intestine, we further evaluated the expression of adiponectin and leptin in the jejunum of mice infected with *T. spiralis* at different time points using quantitative real-time PCR analysis. Following infection for 7 days, the mRNA expression of adiponectin, but not leptin, was significantly induced (Fig. 1B). The levels of adiponectin in the jejunum were rapidly reduced at 14 days post-infection (Fig. 1B). Interestingly, the induced adiponectin mRNA level was associated with the increased worm number in the small intestine of mice after infection (Fig. 1B). As adiponectin and leptin expression in the intestine at the protein level has been previously reported^46,47^, we measured the protein expression of adiponectin and leptin in small intestine tissue lysate of mice infected with *T. spiralis* using ELISA analysis. We found an enhanced production of adiponectin in tissue lysate of small intestine following 7 and 14 days of *T. spiralis* infection (Fig. 1C). Unlike unchanged level in the leptin transcript, the secretion of leptin was reduced in small intestine tissue lysate upon *T. spiralis* infection (Fig. 1C). Altogether, these results indicate that *T. spiralis* infection reduces leptin expression both locally in the small intestine and systemically, but induces intestinal adiponectin expression without influencing at the systemic level. Using Spearman’s rank correlation analysis, we revealed a strong positive association between the expression of adiponectin (Fig. 1D), but not leptin (Fig. 1E), at either mRNA or protein levels and worm number in small intestine of mice infected with *T. spiralis* for 7 days. Although we cannot exclude the possible role of serum leptin in regulating immunity *to T. spiralis* infection, we suggest the potential involvement of adiponectin expression in the small intestine in response to *T. spiralis* infection.

### IL-25 signaling modulates adiponectin expression within the small intestinal tissue

**Figure 2:**
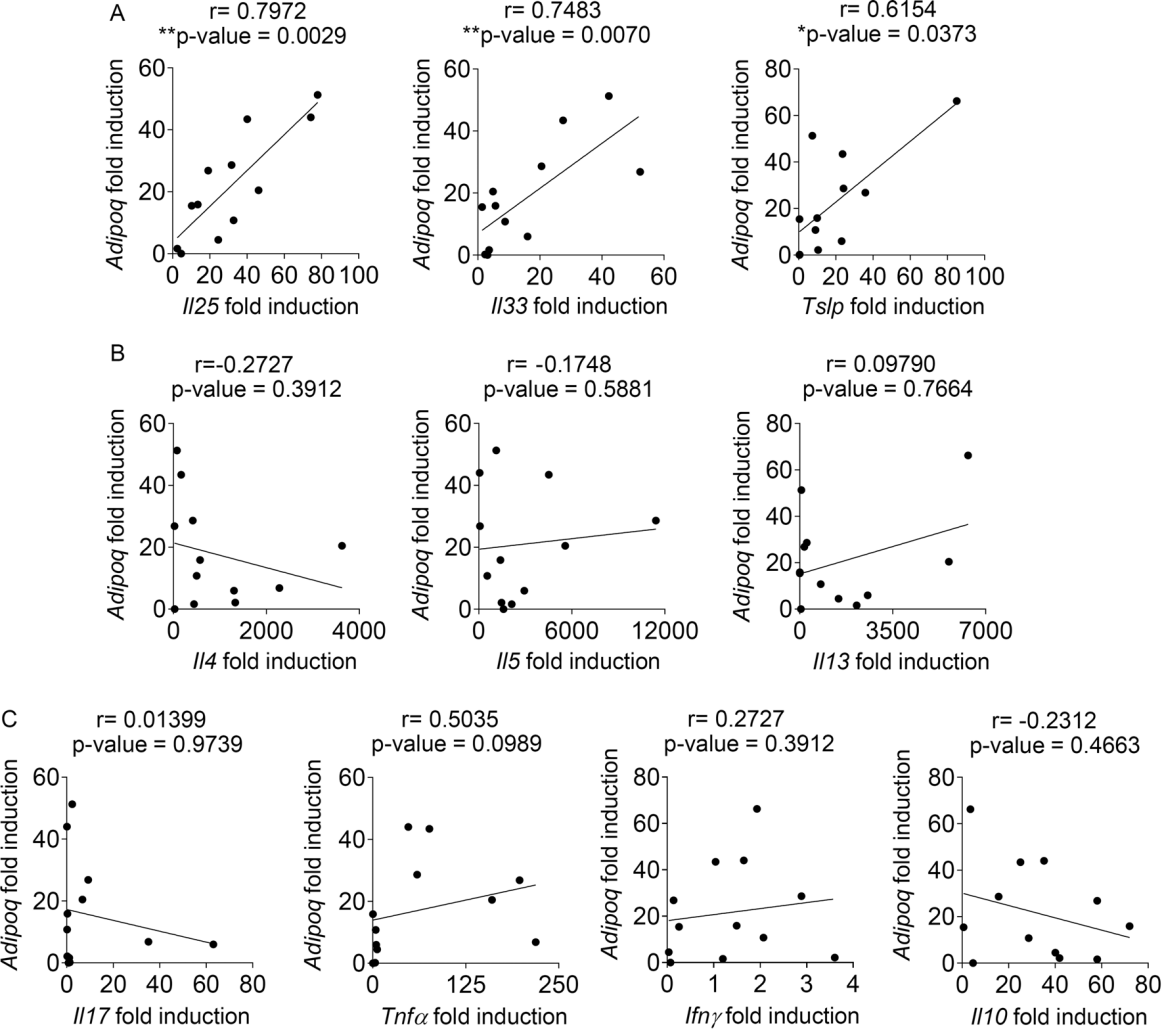
The induction of adiponectin is associated with epithelial cell-derived cytokines in intestinal tissue upon *T. spiralis* infection. (**A**–**C**) *T. spiralis* larvae were orally gavaged into BALB/c mice. Jejunum tissue was collected at 7 days after infection to evaluate the mRNA expression of adiponectin, epithelial cell-derived cytokines, type 2 cytokine, proinflammatory, and regulatory cytokines using quantitative real-time PCR. The correlation between gene expression level of adiponectin and (**A**) epithelial cell-derived cytokines (*Il25*, *Il33*, *Tslp*), (**B**) type 2 cytokine (*Il4*, *Il5*, *Il13*) and (**C**) proinflammatory cytokine (*Il17*, *Tnfα*, *Ifnγ*) or regulatory cytokine (*Il10*) in jejunum tissue of mice infected with *T. spiralis*, with n = 12 mice was analyzed using Spearman’s rank test. Correlation coefficients (r) and *p* values are provided. (*p < 0.05, **p < 0.01).

A recent report suggested that unidentified secretory factors released from the intestinal epithelial cell were involved in the regulation of adiponectin^49^. Since helminth infection up-regulated several cytokines related to type-2 immune responses^6^, we tested the possibility that these cytokines might regulate adiponectin expression in the intestinal tissue during helminth infection. We evaluated the mRNA expression level of epithelial cell-derived cytokines that known to initiate type 2 immune response (*Il25*, *Il33*, *Tslp*), signature type 2 cytokines (*Il4*, *Il5*, *Il13*), proinflammatory cytokines (*Il17*, *Tnfα*, *Ifnγ*), and regulatory cytokine (*Il10*) in jejunal tissues of mice infected with *T. spiralis* for 7 days using quantitative real-time PCR. The association between the expression levels of these cytokines and adiponectin was analyzed using a Spearman’s rank correlation analysis. Among these cytokines that were analyzed (Fig. 2A–C), we found a strong positive correlation between the mRNA expression levels of adiponectin and epithelial cell-derived cytokines *Il25* (r = 0.7972, p = 0.0029), *Il33* (r = 0.7483, p = 0.0070), and *Tslp* (r = 0.6154, p = 0.0373) (Fig. 2A). These data suggest that the intestinal epithelial cell-derived cytokines that are induced by helminth infection may modulate the expression of adiponectin in the intestine.

**Figure 3:**
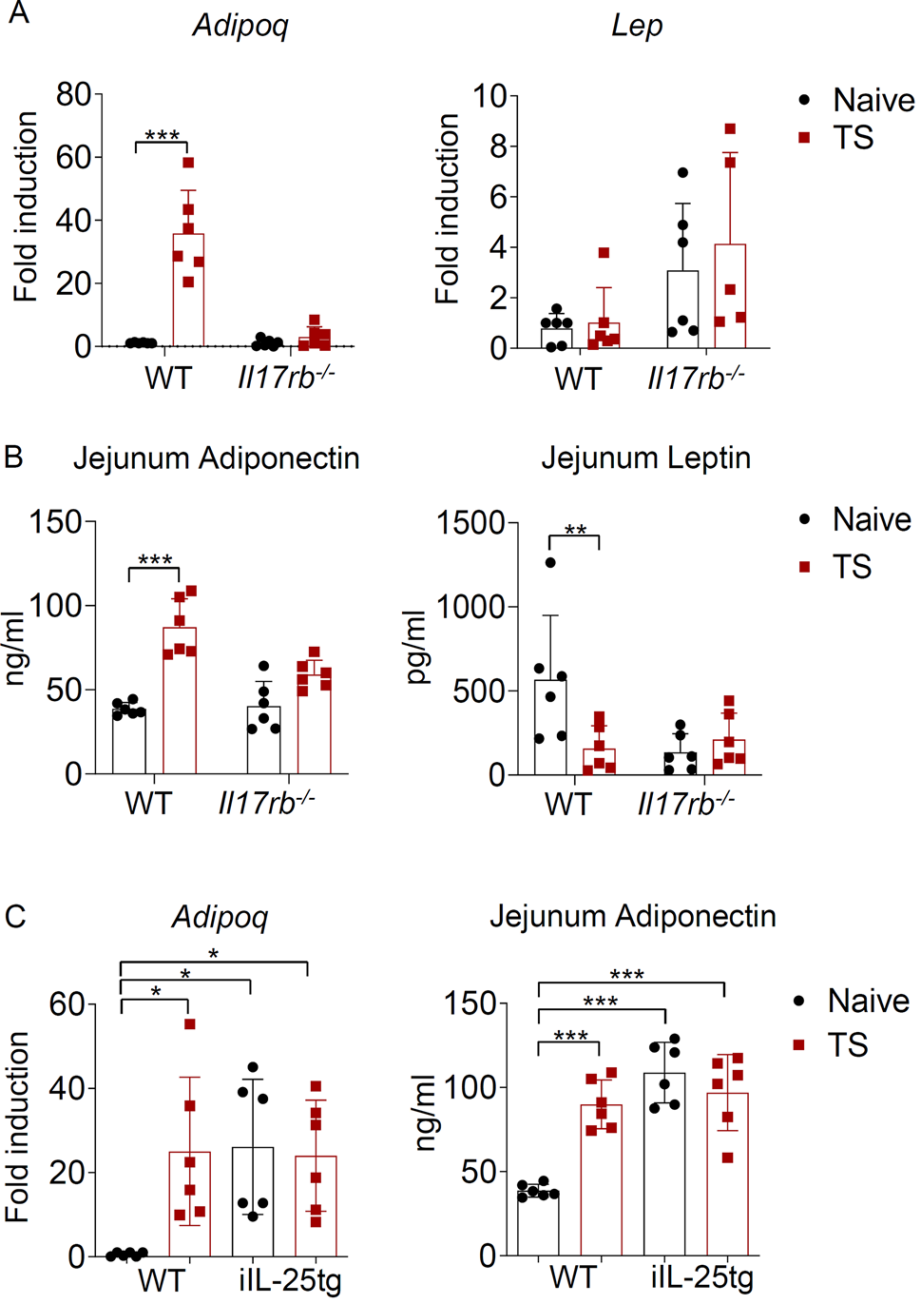
IL-25 signaling is required to induce adiponectin expression in the intestine during *T. spiralis* infection. (**A**,**B**) Wild-type and *Il17rb*^*−*/*−*^ mice were infected with *T. spiralis* larvae by oral gavage. Jejunum tissue was collected at 7 days after infection to evaluate the expression of adiponectin and leptin using quantitative real-time PCR and ELISA analysis. (**A**) The mRNA expression of adiponectin and leptin in the jejunum tissue from uninfected or *T. spiralis*-infected wild-type and *Il17rb*^*−*/*−*^ mice. Data are presented as fold induction over actin (*Actb*) expression, with the mRNA expression levels in uninfected wild-type mice set as 1. (**B**) ELISA analysis of adiponectin and leptin in jejunum tissue lysate from uninfected or *T. spiralis*-infected wild-type and *Il17rb*^*−*/*−*^ mice. Graphs depict individual mice and mean ± SD is representative of three pool independent experiments, with n = 6 mice per group. Significance was determined using two-way ANOVA followed by Bonferroni’s post-test analysis (**p < 0.01, ***p < 0.001). (**C**) Wild-type and iIL-25Tg mice were orally gavaged with 400 larvae of *T. spiralis*. At 7 days of infection, mice were euthanized and jejunum tissue was collected for measurement of adiponectin and leptin expression. (left) Quantitative real-time PCR analysis of adiponectin, Data are presented as fold induction over actin (*Actb*) expression, with the mRNA expression levels in uninfected wild-type mice set as 1 and (right) ELISA analysis of adiponectin in jejunum tissue lysate from uninfected or *T. spiralis*-infected wild-type and iIL-25Tg mice. Graphs depict individual mice and mean ± SD is representative from three pooled independent experiments, with n = 6 mice per group. Significance was determined using one-way ANOVA followed by Turkey post hoc analysis (*p < 0.05, ***p < 0.001).

Because IL-25 expression in the intestinal tissue of *T. spiralis*-infected mice was most strongly associated with the level of adiponectin, we tested the possibility that IL-25 signaling in the intestine induced by helminth infection might regulate adiponectin expression. The expression of adiponectin in the intestinal tissue of IL-25 receptor knockout (*Il17rb*^*−*/*−*^) mice that lack endogenous IL-25 signaling was examined and compared with that of wild-type mice after infection with *T. spiralis* for 7 days. While *T. spiralis* infection enhanced intestinal adiponectin mRNA expression in wild-type mice, there was no induction of adiponectin transcript in *Il17rb*^*−*/*−*^ mice (Fig. 3A). Notably, there was no difference in the mRNA expression level of leptin in small intestinal tissue of either wild type or *Il17rb*^*−*/*−*^ mice upon *T. spiralis* infection (Fig. 3A). Consistent with the mRNA expression data, an increased secretion of adiponectin, but not leptin, was observed in the intestinal tissue lysates of *T. spiralis*-infected wild type mice, but not in those of *Il17rb*^*−*/*−*^ mice (Fig. 3B). We further confirmed the involvement of intestinal IL-25 expression in regulating adiponectin expression using mice overexpressing IL-25 specifically in the epithelial cells of the mucosal layer of the small intestine tissue (iFABP-IL-25Tg mice)^50^. While we observed the induced adiponectin expression only upon *T. spiralis* infection in wild-type mice, naïve iIL-25Tg mice exhibited high level of adiponectin expression and secretion in the intestinal tissue without further induction upon infection (Fig. 3C). Altogether, these data suggest that the epithelial cell-derived cytokines induced by *T. spiralis* infection may play role in regulating intestinal adiponectin expression and IL-25 signaling is among these factors that influences the expression of adiponectin in the intestine.

### Adiponectin is mainly derived from the intestinal epithelial cells during *T. spiralis* infection

**Figure 4:**
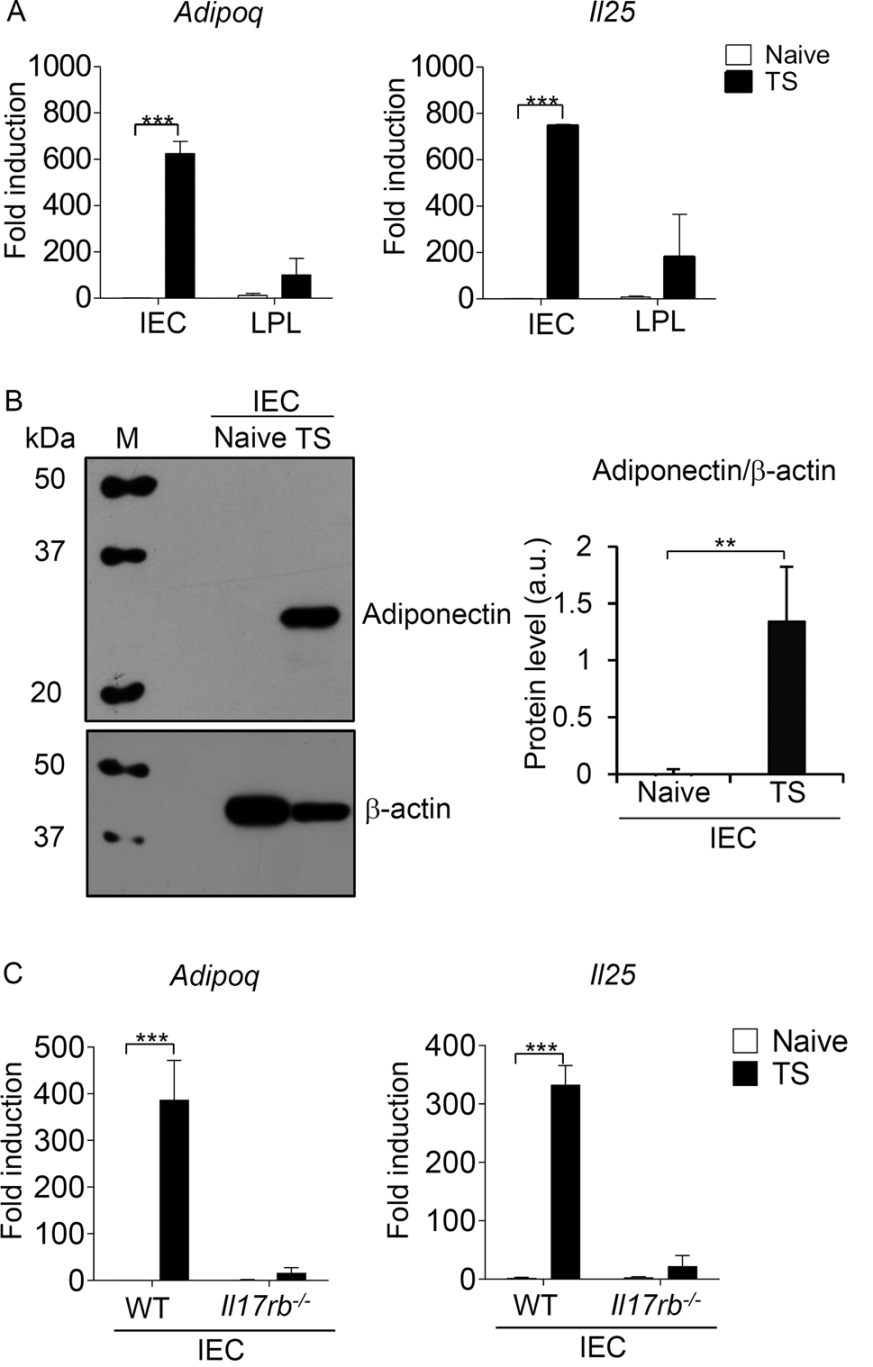
Adiponectin is mainly expressed by intestinal epithelial cells during *T. spiralis* infection. (**A**,**B**) BALB/c mice were orally gavaged with 400 larvae of *T. spiralis*. At 7 days post-infection, mice were sacrificed and the small intestines were harvested. Intestinal epithelial cells (IEC) or lamina propria cells (LPL) were isolated for the assessment of adiponectin and IL-25 expression using quantitative real-time PCR and western analysis. (**A**) Quantitative real-time PCR analysis of *Adipoq* and *Il25* mRNA expression in isolated intestinal epithelial cells and lamina propria cells from uninfected and *T. spiralis*-infected mice. Data are presented as fold induction over actin (*Actb*) expression, with the mRNA expression levels from naive mice set as 1. (**B**) Western blot analysis of adiponectin and β-actin in isolated intestinal epithelial cells from uninfected and *T. spiralis*-infected mice. Lane M represents molecular weight protein marker (kDa). The molecular weights of adiponectin and β-actin are 27 kDa and 42 kDa, respectively. Bar graph represents the protein level by measuring intensity of protein bands in arbitrary units. the intensity of β-actin was normalized with the intensity of adiponectin. The full-length blots are presented in Supplementary Fig. S1. (**C**) Wild-type and *Il17rb*^*−*/*−*^ mice were infected with *T. spiralis* for 7 days and the intestinal epithelial cells were isolated and analyzed for the quantification of adiponectin and IL-25 expression using quantitative real-time PCR. Data are presented as fold induction over actin (*Actb*) expression, with the mRNA levels in intestinal epithelial cells from naïve wild-type mice set as 1. Graphs depict mean ± SD of at least three independent experiments, with n = 3 mice per group. Significance was determined using two-way ANOVA followed by Bonferroni’s post-test analysis and Student’s t-test analysis (**p < 0.01, ***p < 0.001).

Previous studies showed that adiponectin was expressed in one type of intestinal epithelial cells, the Paneth cells in both mice and humans that are regulated by gut Lactobacillus NK6^46^ and in colon intestinal tissue in the subepithelial mesenchymal cells of the lamina propria^45^. To further investigate whether the intestinal epithelial cells could be the main producer of adiponectin in response to helminth infection, we separated a population of intestinal epithelial cells (IEC) and laminar propria cells (LPL) from mice infected with *T. spiralis* for 7 days and evaluated the adiponectin expression using quantitative real-time PCR and western blot analysis. Indeed, the *Adipoq* mRNA was predominantly expressed in the isolated intestinal epithelial cells, but not in the laminar propria cells of mice infected with *T. spiralis* (Fig. 4A). Consistent with previous report^2^, *T. spiralis* infection induced the expression of *Il25* mainly in the intestinal epithelial cells (Fig. 4A). To confirm the mRNA expression data of *Adipoq* in the intestinal epithelial cells, we performed western blot analysis and found a significantly induced level of adiponectin protein expression in these cells following *T. spiralis* infection (Fig. 4B). Because IL-25 signaling contributes to the upregulation of the intestinal adiponectin during *T. spiralis* infection, the intestinal epithelial cells were isolated from *Il17rb*^*−*/*−*^ mice and evaluated for the adiponectin expression during helminth infection. Indeed, we found no induction of *Adipoq* and *Il25* expression in the isolated intestinal epithelial cells of *T. spiralis*-infected *Il17rb*^*−*/*−*^ mice (Fig. 4C). These data suggest that the induction of adiponectin in the intestinal tissue following *T. spiralis* infection is mainly derived from the intestinal epithelial cells.

### The influence of crosstalk between IL-25 and adiponectin in the intestinal epithelial cell function

**Figure 5:**
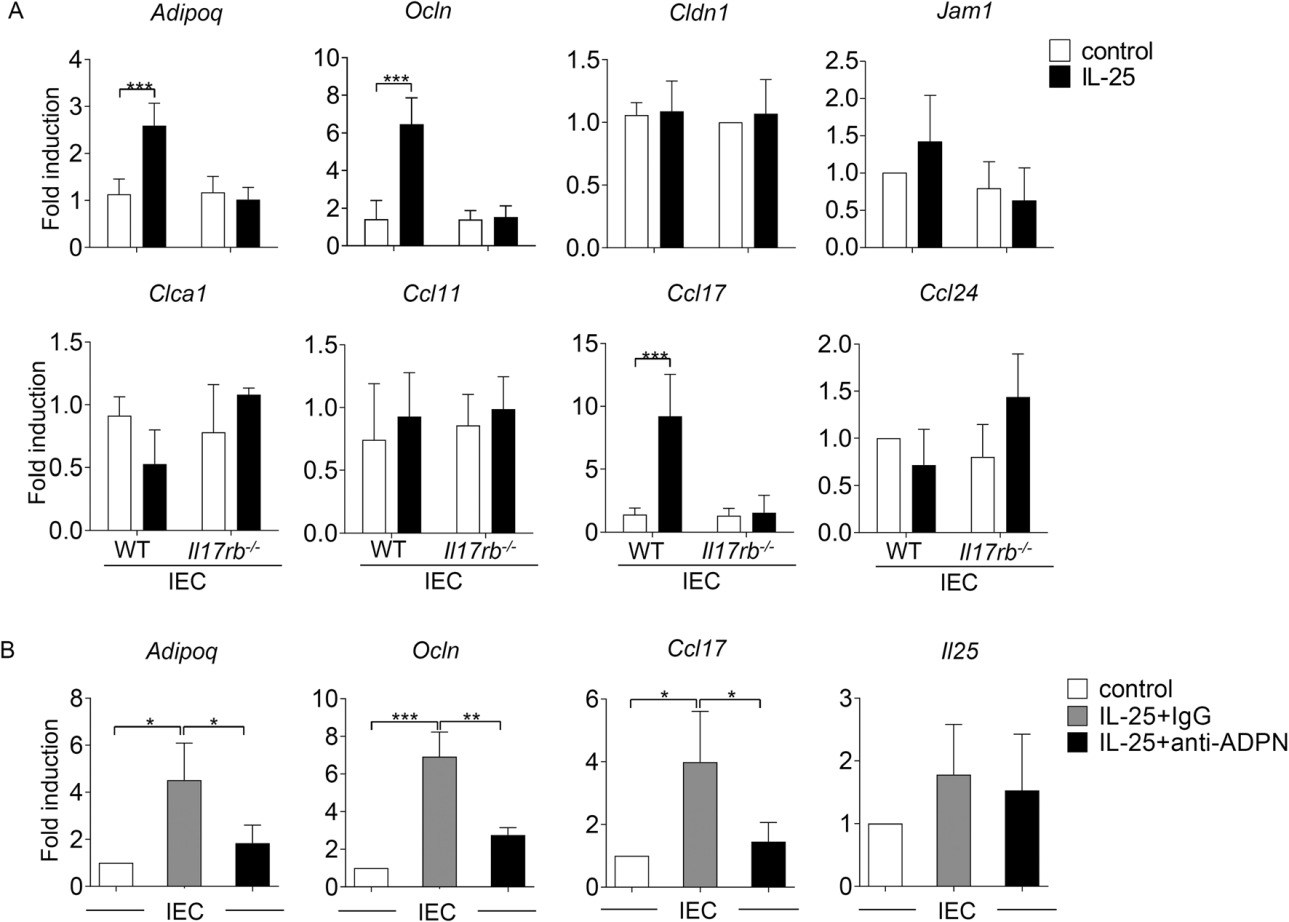
IL-25-induced adiponectin expression promotes the upregulation of occludin and CCL17 in isolated intestinal epithelial cells. Isolated intestinal epithelial cells from wild-type and *Il17rb*^*−/−*^ mice were treated in the presence or absence of IL-25 for 36 h and analyzed for the expression of indicated genes using quantitative real-time PCR. (**A**) Quantitative real-time PCR analysis of adiponectin (*Adipoq*), tight junction-associated genes (*Ocln, Cldn1, Jam1*), mucus-associated genes (*Clca1*), and chemokines (*Ccl11*, *Ccl17*, *Ccl24*). Data are presented as fold induction over actin (*Actb*) expression, with the mRNA levels in the control untreated cells set as 1. (**B**) The isolated intestinal epithelial cells from wild-type mice were treated with IL-25 (100 ng/mL) in the presence of adiponectin neutralizing antibody (anti-ADPN, 10 μg/mL) or isotype control (10 μg/mL) for 36 h. and analyzed for the expression of *Adipoq*, *Ocln, Ccl17*, and *Il25* using quantitative real-time PCR. Data are presented as fold induction over actin (*Actb*) expression, with the mRNA levels in the control untreated cell set as 1. Graphs depict mean ± SD of three independent experiments, with n = 3 mice per group. Significance was determined using two-way ANOVA followed by Bonferroni’s post-test analysis and one-way ANOVA followed by Turkey post hoc analysis (*p < 0.05, **p < 0.01, ***p < 0.001).

Because *T. spiralis* infection induced the expression of adiponectin mainly in the intestinal epithelial cells, which was dependent upon the IL-25 signaling, we further investigated the crosstalk between adiponectin and IL-25 signaling in the intestinal epithelial cells. We first analyzed an in vitro effect of IL-25 in the isolated intestinal epithelial cells in inducing the expression of adiponectin and other genes related to the intestinal epithelial function. The intestinal epithelial cells were isolated from wild-type and *Il17rb*^*−*/*−*^ mice and then treated with or without IL-25 for 36 h. The mRNA expression of adiponectin, tight junction-associated gene (*Ocln*, *Cldn1*, *Jam1*), mucus-associated gene (*Clca1*), and chemokines related to type-2 immune response (*Ccl11*, *Ccl17*, *Ccl24*) was assessed using quantitative real-time PCR. Among genes that were analyzed, IL-25 treatment enhanced the mRNA expression of *Adipoq, Ocln* and *Ccl17* in the intestinal epithelial cells isolated from wild-type but not *Il17rb*^*−*/*−*^ mice (Fig. 5A).

To test whether adiponectin that was induced by IL-25 could play role in regulating intestinal epithelial cell response, we determined the effect of IL-25 on the intestinal expression of *Adipoq, Ocln* and *Ccl17* in the presence of neutralizing antibody against adiponectin or isotype control IgG. Treatment with neutralizing antibody against adiponectin did not affect the expression of IL-25; however, obviously suppressed the effect of IL-25 in inducing *Adipoq*, *Ocln* and *Ccl17* mRNA expression in intestinal epithelial cells (Fig. 5B). These results suggest that IL-25-induced adiponectin expression may play roles in regulating intestinal epithelial cell response, possibly by promoting the expression of genes related to tight junction formation and type-2 immunity in the intestine.

### Adiponectin modulates the intestinal barrier integrity during LPS-induced barrier dysfunction

**Figure 6:**
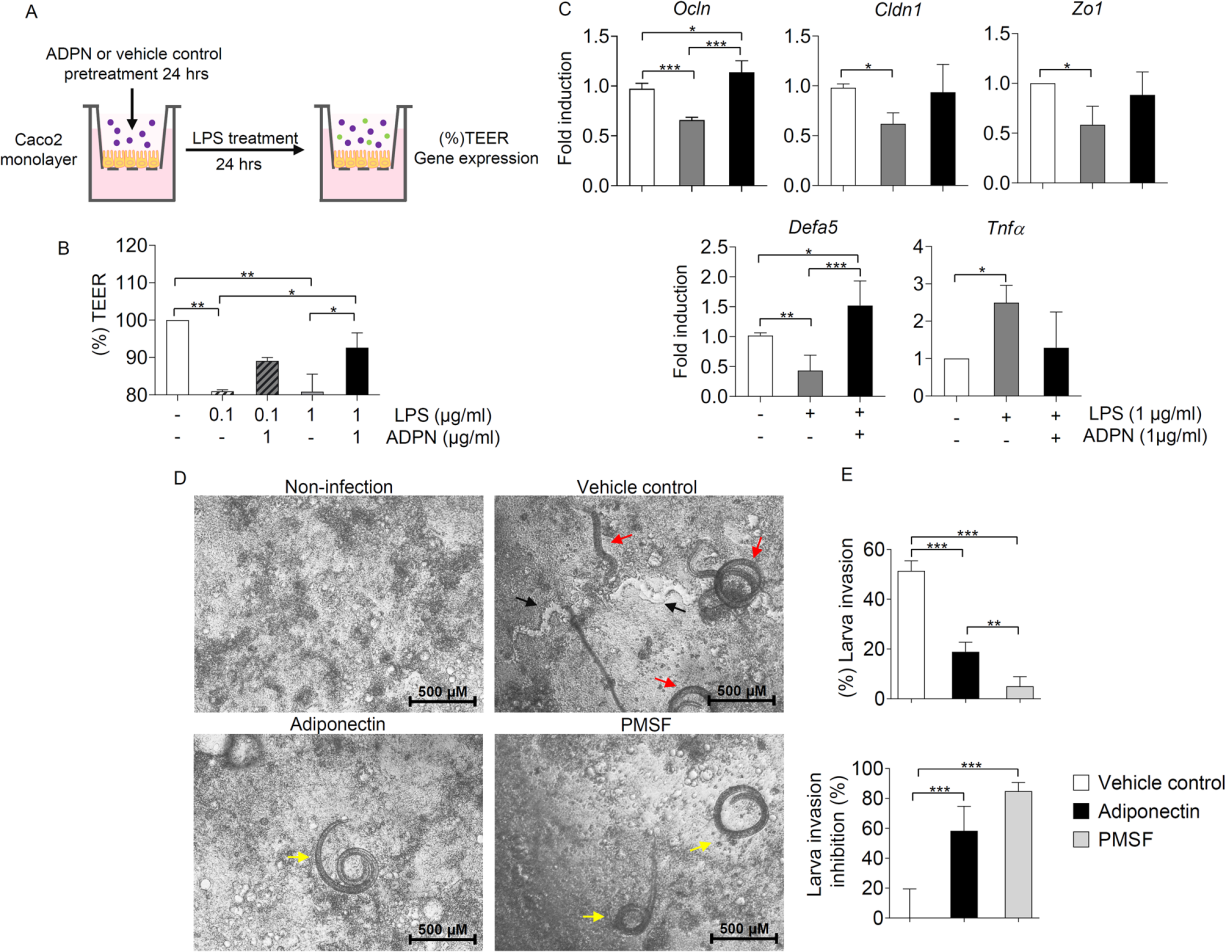
Adiponectin attenuates LPS-induced barrier dysfunction and prevents larval invasion in Caco2 cell monolayers. (**A**–**C**) Caco2 cells were grown in transwell insert of 24-well plate for 21 days. Cells were pre-treated with 1 µg/mL human recombinant adiponectin for 24 h. DMEM with 0.1% FBS was used as vehicle control. Pre-treated cells were then stimulated with 0.1 and 1 µg/mL LPS for 24 h before analysis of barrier integrity and gene expression. (**A**) Transwell system used for investigating the effect of adiponectin in controlling LPS‑induced barrier dysfunction Caco-2 cells. (**B**) (%) TEER values in each condition treatment. (**C**) Quantitative real-time PCR analysis of tight junction-associated genes (*Ocln*, *Cldn1*, *Zo1*), antimicrobial peptides (*Defa5*), and proinflammatory cytokine (*Tnfα*) mRNA expression. Data are presented as fold induction over actin (*Actb*) expression, with the mRNA levels in the control untreated cell set as 1. (**D**,**E**) Caco2 cells were grown in 24-well plate for 21 days. Cells were pre-treated with 1 µg/mL human recombinant adiponectin for 24 h. DMEM with 0.1% FBS was used as vehicle control. In vitro larval invasion assay was performed for 2 h. The PMSF-pretreated ILLs were used as an inhibitor of larval invasion. The pictures of larval invasion were captured under an inverted microscope (Scale bar 500 μm) and the number of invaded larvae and non-invaded larvae were counted under an inverted light microscope. (**D**) Representative data of invaded larvae (migratory path showed as black arrows, Invaded larvae penetrate in cell monolayers show as red arrow) and non-invaded larvae (their coiled on the cell monolayer without movement show as yellow arrow) at 2 h after infection from different treatment groups, including non-infection, Caco2 cells pre-treated with vehicle control, Caco2 cells pre-treated with adiponectin, and Caco2 cells with ILLs that pre-treated with PMSF. (**E**) (%) Larva invasion (above) and (%) Larva invasion inhibition (below) from different treatment groups. Graphs depict mean ± SD of three independent experiments. Significance was determined using one-way ANOVA followed by Turkey post hoc analysis (*p < 0.05, **p < 0.01, ***p < 0.001).

As tight junction-associated gene occludin was induced by adiponectin, we further evaluated whether adiponectin could improve intestinal barrier integrity using an in vitro model of LPS-induced barrier dysfunction^51^. Human colon intestinal epithelial cell line, Caco2 cells were grown in Transwell insert of 24-well plate for 21 days and then pretreated with vehicle control or recombinant adiponectin for 24 h. These cells were then stimulated with 0.1 and 1 µg/mL LPS for 24 h and the value of transepithelial electrical resistance (TEER) was measured (Fig. 6A). Treatment with LPS (both 0.1 and 1 µg/mL) resulted in a reduction of the percentage of TEER (Fig. 6B), indicating that LPS treatment caused destruction of intestinal barrier and increased cell permeability. Interestingly, adiponectin-pretreated cells stimulated with LPS significantly increased percentage of TEER (Fig. 6B), suggesting that adiponectin counteracts the LPS-induced barrier dysfunction. We further determined the effect of adiponectin in alleviating the LPS-induced barrier dysfunction by assessing the mRNA expression of tight junction-associated genes (*Ocln*, *Cldn1*, *Zo1*), antimicrobial peptides (*Defa5*), and proinflammatory cytokine (*Tnfa*) using quantitative real-time PCR. Indeed, LPS treatment significantly decreased the mRNA expression of tight junction-associated genes (*Ocln*, *Cldn1*, *Zo1*) and antimicrobial peptides (*Defa5*); but increased *Tnfa* mRNA expression. Among these genes, pretreatment with adiponectin effectively restored the effect of LPS in reducing the mRNA expression of *Ocln* and *Defa5* but not *Cldn1* and *Zo1* (Fig. 6C). These data suggest that adiponectin may improve the intestinal barrier integrity by regulating epithelial tight-junction barrier.

### Adiponectin protects *T. spiralis* larval invasion of the intestinal epithelial cells in vitro

Previous studies suggested that intestinal epithelial cells are the first line of defense against invading helminth parasites^52^. Because we found that adiponectin prevented the destruction of intestinal epithelial cells by LPS stimulation, we further investigated the effect of adiponectin in protecting *T. spiralis* larval invasion of the intestinal epithelial cells using the in vitro model of larval invasion into Caco2 cells. Caco2 cells were pretreated with adiponectin 24 h before performing an in vitro larval invasion assay; and the invasion and migratory path of larvae were then visualized in cell monolayer. The percentage of larval invasion and larval invasion inhibition was then calculated. In the vehicle control group, we detected larvae invasion and their migratory trace on cell monolayer (Fig. 6D,E). Consistent with previous report^53^, PMSF, a serine protease inhibitor strongly inhibits *T. spiralis* larval invasion into host epithelial cells (Fig. 6D,E). Interestingly, we observed non-invaded larvae coiled on cell monolayer without movement and almost no migratory trace in cells with adiponectin pretreatment (Fig. 6D). Pretreatment of adiponectin thus resulted in the inhibition of larva invasion (Fig. 6E). Altogether, these data suggest that adiponectin may enhance the intestinal barrier integrity; and thereby prevents larval invasion of the intestinal epithelial cells.

### The effect of adiponectin on host response to helminth infection

**Figure 7:**
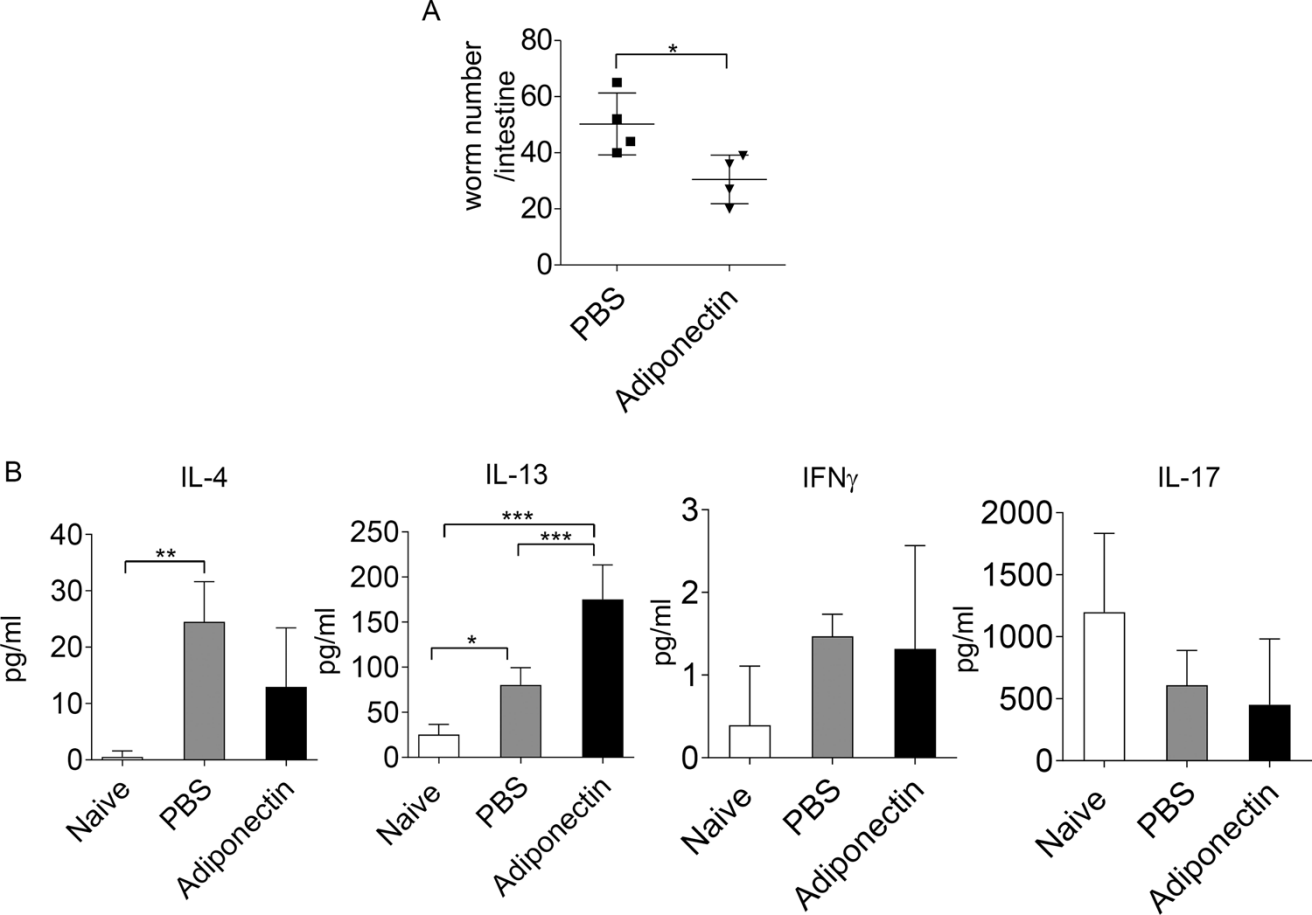
Administration of adiponectin induced intestinal IL-13 secretion and promoted worm expulsion. BALB/c mice were orally gavaged with 400 larvae of *T. spiralis* and given intraperitoneally with recombinant adiponectin (20 µg/mouse) daily for six consecutive days. PBS sterile (pH 7.2–7.4) was injected as vehicle control. At 7 days after infection, mice were sacrificed. (**A**) The whole intestines were collected and subjected for worm burden analysis. (**B**) Jejunum tissue lysate of uninfected or *T. spiralis*-infected mice treated with recombinant adiponectin or PBS at 7 days post-infection was measured for the secretion levels of IL-4, IL-13, IFNγ and IL-17 using ELISA analysis. Graphs depict mean ± SD is representative from two pooled independent experiments, with n = 4 mice per group. Significance was determined using Student’s t-test analysis and one-way ANOVA followed by Turkey post hoc analysis (*p < 0.05, **p < 0.01, ***p < 0.001).

Our above data demonstrated the effect of adiponectin in preventing larvae invasion into epithelial cells in vitro, we further determined whether adiponectin may regulate host responses to *T. spiralis* infection in vivo. We performed an intraperitoneal administration of recombinant adiponectin into BALB/c mice during *T. spiralis* infection and evaluated intestinal worm number as well as cytokine production in intestinal tissue lysates at 7 days post-infection. Interestingly, infected mice treated with recombinant adiponectin, compared to those treated with PBS, exhibited significantly reduced worm number in the intestines (Fig. 7A). Consistent with previous report^2^, *T. spiralis* infection enhanced the production of IL-4 and IL-13 in the intestine (Fig. 7B). Interestingly, administration of adiponectin did not change the levels of IL-4, IFN-γ, and IL-17 secretion; however significantly induced the secretion of IL-13 in small intestinal tissue lysate (Fig. 7B). These data indicate that exogenous administration of adiponectin promotes *T. spiralis* worm expulsion and enhance intestinal host immune response to *T. spiralis* infection.

## Discussion

Recent studies revealed the association between helminth infection and altered levels of adipokine adiponectin and leptin^22–26^; however, the involvement of these adipokines in regulating host response to helminth infection has not yet been elucidated. In the present study, we found that *T. spiralis* helminth infection induced adiponectin, but not leptin expression in the intestinal tissue. The intestinal adiponectin expression induced by *T. spiralis* infection was mainly derived from the intestinal epithelial cells and regulated by IL-25 signaling. We further demonstrated the role of adiponectin in regulating the intestinal epithelial cells to enhance tight junction gene occludin and Th2-attracting chemokine CCL17, attenuating barrier destruction by LPS treatment, and inhibiting larva invasion into intestinal epithelial barrier of Caco-2 cell monolayers in vitro. Treatment with adiponectin in vivo effectively reduced intestinal worm burden and induced intestinal IL-13 expression.

Accumulating evidence showed that there was a change in the circulating level of leptin and adiponectin during infection with the intestinal helminth parasites^22–26^. In this study, we found that serum adiponectin remained unchanged but leptin was reduced following *T. spiralis* infection, resulting in an increased serum adiponectin/leptin ratio. It has been suggested that decreased circulating levels of leptin during *T. spiralis* helminth infection may be caused by the reduction of food intake during helminth infection^23^. Indeed, intraperitoneal injection of recombinant leptin to replenish the baseline of leptin level during *T. spiralis* infection resulted in delayed worm expulsion in the intestines and reduced antigen-specific IL-4 and IL-13 production^23^. However, it remains unclear whether or not the increased ratio of adiponectin and leptin in the circulation following helminth infection may involve in the consequence of helminth infection on an improved metabolic function. Unlike unchanged adiponectin level in the serum, we found an elevated adiponectin levels in the intestinal tissue lysates of *T. spiralis*-infected mice. Indeed, the expression of adiponectin in the gut during helminth infection is poorly understood. Previous report showed that mouse intestinal epithelial cells can express adiponectin that can be modulated by the intestinal microbiota^46^. Because we found the strong association between the expression of the epithelial cell-derived cytokines IL-25, IL-33, TSLP and adiponectin during *T. spiralis* infection, it is likely that these epithelial cell-derived cytokines induced by *T. spiralis* infection can regulate the expression of adiponectin in the intestinal tissue. Indeed, deficiency of IL-25 receptor and overexpression of IL-25 cytokine in the intestinal epithelial cells influenced the intestinal adiponectin expression. It is possible that other epithelial cell-derived cytokines IL-33 and TSLP may also have similar function in regulating adiponectin expression. Further study is required to elucidate the functional relationship between these epithelial cell-derived cytokines and adiponectin expression and the potential linkage to the change of intestinal microbiota during infection.

Because IL-25 signaling was required for the induction of adiponectin in the intestinal epithelial cells, the effect of adiponectin neutralization during IL-25 treatment on isolated-intestinal epithelial cells was evaluated. Indeed, IL-25 induced occludin and CCL17 mRNA expression relying on the expression of adiponectin. Thus, the effect of adiponectin in promoting intestinal barrier integrity was evaluated using the LPS-induced barrier dysfunction model of the human intestinal epithelial Caco-2 cell line. We found that adiponectin could protect the LPS-induced decrease of TEER and augment occludin and α-defensin 5 expression, suggesting its ability to prevent intestinal barrier dysfunction. Recent study indicated that administration of α-defensin 5, a potent antimicrobial peptide secreted from intestinal mucosal epithelial cells, was able to suppress ethanol and colitis-induced barrier dysfunction and inflammation in the small intestine^54^. In addition, using an in vitro model of the larvae invasion of the intestinal epithelial Caco2 cells, we found the protective effect of adiponectin in controlling the infective larvae to invade the intestinal epithelial cell monolayers. Although these data suggest that adiponectin expression that was induced during helminth infection may directly promote the barrier function of the intestinal epithelial cells through enhancing tight junction barrier, the underlying mechanism by which adiponectin promotes intestinal barrier function remains unclear. Recent finding demonstrated that LPS induced barrier disruption by downregulating tight junction proteins expression, including ZO-1, occludin, and claudin-1 in Caco2 cells through enhancing the activation of NF-κB, PI3K/AKT, and P38 pathways^55^. Moreover, previous study indicated that the excretory secretory (ES) products derived from *T. spiralis* directly modulated barrier destruction of Caco2 by reducing the expression of ZO-1, occludin, and claudin-1 through the P38-MAPK signaling pathway^56^. Further studies are needed to evaluate the possibility that adiponectin might play role in maintaining intestinal barrier integrity through attenuating the activation of NF-κB, PI3K/AKT, and P38 pathways.

Consistent to the effect of adiponectin in vitro, adiponectin treatment in vivo during *T. spiralis* infection reduced intestinal worm numbers. Treatment of adiponectin during infection did not affect IFNγ and IL-17 cytokine production but enhanced IL-13 secretion levels in small intestinal tissues. Related studies suggest that adiponectin can modulate immune response by suppressing IFNγ and IL-17 cytokine production in high-fat diet mice^31^. The functions of adiponectin might be varied depending on their isoforms, target cell types, and disease models. During helminth infection, it is likely that adiponectin may promote worm expulsion by enhancing type-2 cytokine production in the intestine and also strengthening the intestinal epithelial cell barrier. The contribution of adiponectin in modulating type-2 associated immune cell response at the site of infection in vivo will likely be investigated in the future. As IL-25 was reported to play important roles in promoting immunity to *T. spiralis*^2^, it is likely that *T. spiralis* infection induces intestinal epithelial cells to produce IL-25 that enhances the expression of adiponectin, thus cooperates to promote immunity to *T. spiralis* infection.

In summary, this study proposed the additional roles of adipokines in regulating intestinal host response to helminth infection. The expression of adiponectin in the intestinal tissue was induced and regulated by the epithelial cell-derived cytokine IL-25 upon *T. spiralis* infection. Adiponectin expression influences the function of IL-25 on inducing the expression of tight junction gene occludin and type-2 associated chemokine CCL17 in the intestinal epithelial cells. Treatment of adiponectin protected barrier destruction by LPS stimulation and prevented helminth larvae invasion in vitro; and promoted worm clearance and type-2 cytokine secretion in the intestine in vivo. These findings provide more understanding of the regulation and function of adiponectin in the intestinal tissue during helminth infection. Furthermore, it will be interesting to further analyze the tissue function of adiponectin and other adipokines in regulating immune response at the tissue site of other disease models.

## Materials and methods

### Animals

Male or female BALB/c and female ICR mice, 6- to 8-week-old, used in all experiments were obtained from Nomura Siam International Co., Ltd., Thailand. All genetically modified iFABP-IL-25 transgenic and *Il17rb*^−/−^ mice were bred and housed under specific pathogen-free conditions in the animal facility of Thammasat University. As described previously^50^, the genotyping primers of iIL-25 transgenic mice were as follows: forward primer (5′-CAGGACCGAATCTCTGCTTT-3′) and reverse primer (5′-TCAAGTCCCTGTCCAACTCA-3′); and the genotyping primers of *Il17rb*^−/−^ mice were as follows: WT forward primer (5′-GAGTCAGCCAAATAAGCTTTTGA-3′), Neo forward primer (5′-GGGCTCTATGGCTTCTGA-3′), and common reverse primer (5′-TGATTAAGGGTTCTTTGCCAGTA-3′). The Thammasat University Animal Care and Use Committee approved all animal procedures (008/2562). All animal experiments were performed by the relevant guidelines and regulations and carried out in compliance with the ARRIVE guidelines (https://arriveguidelines.org).

### Parasite infection

*Trichinella spiralis* (ISS62)^57^ originated from an outbreak in Mae Hong Son Province in 1986 and was maintained in ICR mice. The larvae were recovered from muscle of infected mice after 30 days post-infection by pepsin-acid digestion. Mice were orally gavaged with 400 *T. spiralis* larvae and euthanized at indicated time points after infection. To investigate the role of adiponectin in mediating immunity to *T. spiralis* infection, BALB/c mice were infected with 400 *T. spiralis* larvae and recombinant mouse adiponectin (20 µg/mouse, Peprotech) were given intraperitoneally daily for six consecutive days. PBS sterile (pH 7.2–7.4) was injected as vehicle control (4 mice/group). At 7 days post-infection, mice were euthanized by CO_2_ inhalation.

### Worm burden assessment

To enumerate the intestinal worm burden of *T. spiralis*-infected mice treated with recombinant mouse adiponectin or PBS, the worm burden assessment was performed as previously described^58^. Briefly, the small intestines were harvested, opened longitudinally, and incubated in Hanks’ Balanced Salt Solution (HBSS) with shaking at 200 rpm for 2 h at 37 °C. After incubation, the small intestines were agitated. The worm numbers were then be counted using an inverted microscope.

### Intestinal epithelial cell (IEC) and lamina propria (LP) cell isolation

Intestinal epithelial cell and lamina propria cell isolation was performed as previously described with some modifications^58,59^. Briefly, the small intestines were harvested, and mesentery, adipose tissue, and payer’s patches were carefully removed. The small intestines were then opened longitudinally, cut into small pieces with 1 cm, and washed with cold HBSS, followed by vigorously shaking in HBSS with 10% heat-inactivated fetal bovine serum (FBS), 20 μM Hepes, 5 mM EDTA, 1% penicillin–streptomycin (P/S), 1 mM DTT, for 20 min at 37 °C. The releasing epithelial cells were incubated in HBSS containing 0.6 U/mL Dispase and 10 μg/mL DNaseI for 10 min at 37 °C. After digestion, the tissue-enzyme mixture was centrifuged at 1000×*g* for 5 min at 4 °C. The pellet was resuspended in 10 mL HBSS containing 10% FBS, 1%P/S before filtering through nylon mesh sterile. For lamina propria cell isolation, the small intestinal tissues were washed with HBSS and digested with 1 mg/mL collagenase A (Roche, Mannheim, Germany), 0.05 mg/mL Liberase, and 0.125 mg/mL DNase I (Roche) at 37 °C for 30 min. Digested cells were then subjected to Percoll centrifugation (37.5%), and LP leukocytes were isolated from the interface, and after washing, resuspended in complete DMEM containing 10% heat-inactivated FBS and 1% penicillin–streptomycin, filtered with nylon mesh sterile for further analysis. The purity and the phenotype of each cell types, isolated epithelial cells and lamina propria cells were verified by flow cytometry as previously described^60^. In some experiments, intestinal epithelial cells were treated with or without IL-25 as prepared in complete DMEM (100 ng/mL, R&D system) for 36 h. Endotoxin levels in recombinant mouse IL-25 were verified by the company and certified to be lower than < 0.01 EU/μg protein, as measured by the Limulus Amebocyte Lysate (LAL) method. Anti-mouse adiponectin antibody (10 μg/mL, AF119; R&D system) was used for neutralization of adiponectin.

### Cell culture of intestinal epithelial cell line Caco-2

Human colon intestinal epithelial cell lines (Caco-2 cells, ATCC HTB-37) were cultured in Dulbecco’s modified eagle medium (DMEM, Gibco™) with high glucose supplemented with 10% heat-inactivated FBS and 1% P/S and maintained at 37 °C in a humidified chamber of 5% CO_2_ as previously described^49^. Confluent cells (85–90%) were used for further investigation.

### LPS‑induced barrier dysfunction

The effect of adiponectin in controlling LPS‑induced barrier dysfunction was performed as previously described with some modifications^51^. Caco2 cells were plated at 5 × 10^4^ cells/well in transwell insert of 24-well plate (0.3 cm^2^, 0.4 µm pore size). Cells were changed with the medium every 2 days and cultured for 21 days until reaching full confluence. Trans-epithelial electrical resistance (TEER) was measured to determine the integrity of cell monolayers using Millicell ERS-2 Voltohmmeter (Merck). The TEER values between 400 to 500 Ω cm^2^ were acceptable for further investigation. Cells were then pre-treated with 1 µg/mL human recombinant adiponectin (Peprotech) for 24 h^61^. DMEM with 0.1% FBS was used as vehicle control. After incubation, cells were added with 0.1 and 1 µg/mL LPS for 24 h and the TEER values were assessed and calculated as the percentage of unit area resistance of different treatment groups compared to control untreated cells. For quantification of gene expression, cells were washed with DPBS twice, harvested for RNA extraction and determined for gene expression using quantitative real time-PCR.

### In vitro larval invasion assay

Caco2 cells were plated at 5 × 10^4^ cells/well in a 24-well plate and cultured for 21 days until reaching full confluence. During cultivation, the medium was changed every 2 days. Cells were then treated with 1 µg/mL human recombinant adiponectin (Peprotech) for 24 h. DMEM with 0.1% FBS was used as vehicle control. After incubation, in vitro larval invasion assay was performed as previously described^52,53^. Briefly, muscle larvae were isolated from infected mice at 30 days post-infection by pepsin-acid digestion. Muscle larvae were stimulated with 5% mouse bile for 2 h at 37 °C to induce intestinal infective larvae (IILs). The ILLs were washed thrice with PBS supplemented with 1%P/S. Then 100 ILLs were prepared in serum-free DMEM containing 1.75% agarose, overlaid on cell monolayer, and incubated for 2 h at 37 °C in 5% CO_2_. In addition, the ILLs were activated with 1.25 mM PMSF in serum-free DMEM for 2 h at 37 °C to use as an inhibitor of larval invasion. After incubation, the larval invasion was visualized and counted under an inverted light microscope. The percentage of larva invasion was calculated according to an equation as (%) Larva invasion = (number of invaded larvae/numbers of invaded larvae and non-invaded larvae) × 100%. In contrast, the percentage of larva invasion inhibition was calculated following an equation as Larva invasion inhibition (%) = (number of invaded larvae in different treatment groups − number of invaded larvae in the vehicle control group)/number of invaded larvae in the vehicle control group × 100%.

### RNA extraction/quantitative RT-PCR

Total RNA was isolated using the TRIzol^®^ reagent (Invitrogen) according to the manufacturer’s instructions. Total RNA at 1 ng to 5 µg was used to generate cDNA using oligo-dT, RNaseOut™ Recombinant Ribonuclease Inhibitor (Invitrogen), and MMLV reverse transcriptase (Invitrogen). To quantify target mRNA, cDNA samples were amplified in iTaqTM universal SYRB^®^ Green Supermix (Biorad Laboratories) with the primer listed in Supplementary Table S1 using CFX96 Touch Real-Time PCR Detection System (Biorad Laboratories). Endogenous actin (*Actb*) transcript level was used as an internal control to normalize the expression of target genes.

### ELISA analysis

Jejunum tissues frozen at liquid nitrogen (LN_2_) were thawed and homogenized in modified RIPA buffer (50 mM Tris–HCl pH 7.4, 1% Triton X-100, 0.2% sodium deoxycholate, 0.2% sodium dodecylsulfate (SDS), 1 mM EDTA) containing 1 mM PMSF (Phenylmethanesulfonyl fluoride, Sigma-Aldrich) and protease inhibitor cocktail (Roche). Homogenized jejunum tissue lysates were then used to determine the concentration of cytokines. IL-4, IFNγ, and IL-17 were analyzed using the antibody pairs from BD Pharmingen, while IL-13 was assessed using ELISA kits from R&D Systems. For quantitative measurement of adiponectin and leptin, serum and homogenized jejunum tissue lysates were measured using the adiponectin and leptin ELISA kit from R&D Systems. All cytokine and adipokine ELISA assays were performed according to the manufacturer’s instructions.

### Western blot analysis

For western blot analysis, homogenized jejunum tissue lysates were concentrated using a SpeedVac vacuum concentrator and 30–40 µg protein lysates were then separated by 10% SDS-PAGE (BioRad Laboratories) and electrophoretically transferred to a PVDF (Polyvinylidene fluoride) membrane (Millipore). The membrane was then blocked and incubated with the primary antibodies specific for adiponectin (1:1000, #C45B10, Cell Signaling Technology). After incubation overnight, the membrane was washed and incubated with goat anti-rabbit IgG (heavy and light chain) antibody-conjugated to horseradish peroxidase (HRP) (1:2000, Cell Signaling Technology). Immunoblots were developed with ECL substrate (BioRad Laboratories) and exposed to autoradiography Films (Kodak X-Ray Film, ABM Good). The intensity of the indicated band was detected using Quantity One software and a GS-800TM Calibrated Densitometer (Bio-Rad Laboratories). The quantification of protein was analyzed with Image lab software and normalized using β-actin (1:10,000, #AC-15, Sigma-Aldrich) as an internal control.

### Statistical analysis

Each experiment was carried out at least three times. All data are expressed as mean value ± SD. Data were analyzed using the Student’s t-test analysis, one-way ANOVA with Turkey’s post hoc analysis, and two-way ANOVA followed by Bonferroni’s post-test. Correlation study was analyzed using Spearman’s rank correlation coefficient. Statistical analysis was performed with GraphPad Prism 5 Software. A value of p < 0.05 was considered significant.

### Supplementary Information

Supplementary Information.

## Data Availability

All data have been included in the manuscript and the supplementary information.
