# Supplementary material for: Regulation and function of adiponectin in the intestinal epithelial cells in response to Trichinella spiralis infection
Source: Sci Rep. 2023 Aug 27;13:14004. doi: 10.1038/s41598-023-41377-x (PMC10460792; doi:10.1038/s41598-023-41377-x)
Supplement: Supplementary file 1 — Supplementary Information. [file 41598_2023_41377_MOESM1_ESM.pdf]

**Supplementary Information**

# Regulation and function of adiponectin in the intestinal epithelial cells in response to *Trichinella spiralis* infection

Running title: The role of intestinal adiponectin during *Trichinella spiralis* infection

Siranart Jeerawattanawart[1, 2], Adithap Hansakon[3], Sittiruk Roytrakul[4], Pornpimon Angkasekwinai[1,5*]

[1]Department of Medical Technology, Faculty of Allied Health Sciences, Thammasat University, Pathum Thani 12120 Thailand.

[2]Graduate Program in Biomedical Sciences, Faculty of Allied Health Sciences, Thammasat University, Pathum Thani 12120 Thailand.

[3]Chulabhorn International College of Medicine, Thammasat University, Pathum Thani 12120 Thailand.

[4]Functional Proteomics Technology Laboratory, Functional Ingredients and Food Innovation Research Group, National Center for Genetic Engineering and Biotechnology, National Science and Technology Development Agency, Pathum Thani 12120 Thailand.

[5]Research Unit in Molecular Pathogenesis and Immunology of Infectious Diseases, Thammasat University, Pathum Thani 12120 Thailand.

*** Correspondence:**

Pornpimon Angkasekwinai

upornpim@tu.ac.th, p.akswn@gmail.com

**Supplementary Table S1.** Oligonucleotide primers used for Quantitative real-time PCR.

| Gene | Primer sequence (5'-3') |
|---|---|
| Mouse *Adipoq* | Forward: GAT GCA GGT CTT CTT GGT CCT A<br>Reverse: TTC TCC AGG CTC TCC TTT CC |
| Mouse *Lep* | Forward: CAC ACA CGC AGT CGG TAT C<br>Reverse: TGGTGAGGACCTGTTGATAGA |
| Mouse *Il25* | Forward: CAC ACT GCG TCA GCC TAC AGA<br>Reverse: TGT CGT AAA GTG GGA CGG AGT |
| Mouse *Il33* | Forward: TCC TTG CTT GGC AGT ATC CA<br>Reverse: TGC TCA ATG TGT CAA CAG ACG |
| Mouse *Tslp* | Forward: TCG AGG ACT GTG AGA GCA AGC CAG<br>Reverse: CTG GAG ATT GCA TGA AGG AAT ACC A |
| Mouse *Il4* | Forward: AGA TCA TCG GCA TTT TGA ACG<br>Reverse: TTT GGC ACA TCC ATC TCC G |
| Mouse *Il5* | Forward: CGC TCA CCG AGC TCT GTT G<br>Reverse: CCA ATG CAT AGC TGG TGA TTT TT |
| Mouse *Il13* | Forward: GCT TAT TGA GGA GCT GAG CAA CA<br>Reverse: GCC CAG GTC CAC ACT CCA TA |
| Mouse *Il17* | Forward: CTC CAG AAG GCC CTC AGA CTA C<br>Reverse: GGG TCT TCA TTG CGG TGG |
| Mouse *Tnf*$\alpha$ | Forward: CAG GCG GTG CCT ATG TCT C<br>Reverse: CGA TCA CCC CGA AGT TCA GTA G |
| Mouse *Ifn*$\gamma$ | Forward: GAT GCA TTC ATG AGT ATT GCC AAG T<br>Reverse: GTG GAC CAC TCG GAT GAG CTC |
| Mouse *Il10* | Forward: GGT TGC CAA GCC TTA TCG GA<br>Reverse: ACC TGC TCC ACT GCC TTG CT |
| Mouse *Ocln* | Forward: GAG CTT ACA GGC AGA ACT AGA C<br>Reverse: CAG CCA TGT ACT CTT CAC TCT C |
| Mouse *Cldn1* | Forward: AGG TCT GGC GAC ATT AGT GG<br>Reverse: TGG TGT TGG GTA AGA GGT TG |

| | |
|---|---|
| Mouse *Jam1* | Forward: CAC CGG GTA AGA AGG TCA TTT A<br>Reverse: GAA CCT GTA GCA CCT GAG TAA G |
| Mouse *Claca1* | Forward: GAG TCC CTC ATC CAA CTG AAC<br>Reverse: CCT GAG TCA CCA TGT CCT TTA T |
| Mouse *Ccl11* | Forward: CTG CTT GAT TCC TTC TCT TTC CTA A<br>Reverse: GGA ACT ACA TGA AGC CAA GTC CTT |
| Mouse *Ccl17* | Forward: AGA GCT GCT CGA GCC ACC AAT GTA<br>Reverse: CAC CAA TCT GAT GGC CTT CTT CAC |
| Mouse *Ccl24* | Forward: GGG TCA TCT TCA TCA CCA AGA AGG<br>Reverse: GGT CTG TCA AAC CCC AAA GCA |
| Mouse and Human *Actb* | Forward: GAC GGC CAG GTC ATC ACT ATT G<br>Reverse: AGG AAG GCT GGA AAA GAG CC |
| Human *Ocln* | Forward: CCA ATG TCG AGG AGT GGG<br>Reverse: CGC TGC TGT AAC GAG GCT |
| Humam *Cldn1* | Forward: AAG TGC TTG GAA GAC GAT GA<br>Reverse: CTT GGT GTT GGG TAA GAG GTT |
| Human *Zo1* | Forward: ATC CCT CAA GGA GCC ATT C<br>Reverse: CAC TTG TTT TGC CAG GTT TTA |
| Human *Defa5* | Forward: ACC CAG AAG CAG TCT GGG GAA GA<br>Reverse: GGT GGC TCT TGC CTG AGA ACC TGA |
| Human *Hbd2* | Forward: CCA GCC ATC AGC CAT GAG GGT<br>Reverse: GGA GCC CTT TCT GAA TCC GCA |
| Human *Tnf*$\alpha$ | Forward: CCA GGG ACC TCT CTC TAA TCA<br>Reverse: TCA GCT TGA GGG TTT GCT AC |

*Adipoq,* Adiponectin C1Q And Collagen Domain Containing; *Lep,* Leptin; *Il25*, Interleukin 25; *Il33,* Interleukin 33; *Tslp*, Thymic Stromal Lymphopoietin; *Il4*, Interleukin 4; *Il5*, Interleukin 5; *Il13*, Interleukin 13; *Il17*, Interleukin 17; *Tnfa*, Tumor Necrosis Factor-Alpha; *Ifn*$\gamma$*,* Interferon Gamma; *Il10*, Interleukin 10; *Ocln*, Occludin; *Cldn1*, Claudin 1; *Jam1*, Junctional Adhesion Molecule 1; *Claca1*, chloride channel accessory 1; *Ccl11*, C-C Motif Chemokine Ligand 11;

*Ccl17*, C-C Motif Chemokine Ligand 17; *Ccl24*, C-C Motif Chemokine Ligand 24; *Actb*, β-actin; *Defa5*, α-Defensin 5; *Hbd2*, β-Defensin 2

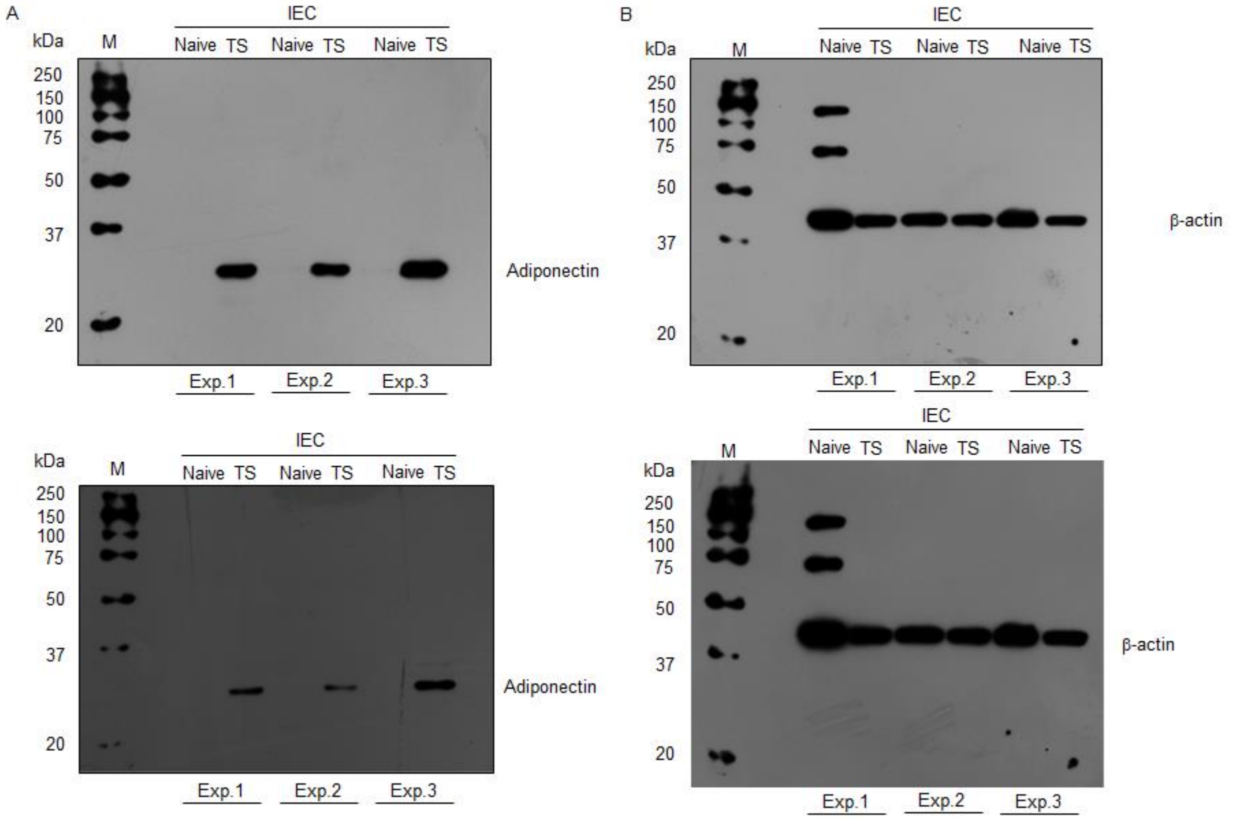


**Supplementary Figure S1.** Full-length blots from Western blot analysis of the adiponectin protein expression in the lysate of intestinal epithelial cells from mice infected with *T.spiralis* for 7 days compared to naive mice. Lane M represents molecular weight protein marker (kDa). The molecular weights of adiponectin and β-actin are 27 kDa and 42 kDa, respectively. The images shown in (A) adiponectin at different exposure for 10 (upper) and 5 (lower) minutes and (B) β-actin at 30 (upper) and 60 (lower) seconds were obtained from the same blot in three independent experiments.
